# Seasonal changes of soil microbiota and its association with environmental factors in coal mining subsidence area

**DOI:** 10.1186/s13568-023-01653-5

**Published:** 2023-12-20

**Authors:** Meng Wang, Mengyao Sun, Yue Zhao, Yuying Shi, Shuo Sun, Shen Wang, Yiping Zhou, Lei Chen

**Affiliations:** 1 Technology Innovation Center of Restoration and Reclamation in Mining induced Subsidence Land, Shandong Provincial Lunan Geology and Exploration Institute (Shandong Provincial Bureau of Geology and Mineral Resources, Ministry of Natural Resources of China, No.2 Geological Brigade), Jining, 272000 China; 2 College of Life Sciences, Qufu Normal University, Qufu, Shandong Province China

**Keywords:** New wetland in coal mining subsidence area, Environmental factors, Soil microbiota, Seasonal variation

## Abstract

As a special type of wetland, the new wetland in the coal mining subsidence area is highly sensitive to environmental changes. In recent years, more and more attention has been paid to the studies of soil microbial diversity in newly born wetlands in coal mining subsidence areas. However, there are few reports on the seasonal variation of soil microbial diversity and its relationship with soil physical and chemical properties. In this study, 16S rRNA gene sequencing technology was used to analyze the seasonal changes of soil microbial composition and functional diversity in newly formed wetlands in coal mining subsidence areas, and to determine the seasonal changes of soil nutrient elements and physical and chemical properties in coal mining subsidence areas, so as to analyze the correlation between soil microbial diversity and soil nutrient elements and physical and chemical properties in newly formed wetlands in coal mining subsidence areas. A total of 16,050 OTUs were obtained after sample gene noise reduction. *Proteobacteria*, *Acidobacteriota* and *Bacteroidota* were the highest abundance in the coal mining subsidence area of Jining. The two seasons gathered separately, and temperature (Temp), total phosphorus (TP), available phosphorus (AP), total organic carbon (TOC) and dry matter content (DMC) were the key factors for the seasonal change of soil microbial community in the wetland of the coal mining subsidence area of Jining. The contents of Temp, AP and TP were significantly correlated with the abundance of soil microorganisms in summer subsidence area, while the contents of DMC and TOC were significantly correlated with the abundance of soil microorganisms in winter subsidence area. Soil microbial diversity in coal mining subsidence area was correlated with the seasons. Temp, TP, AP, TOC and DMC were the key factors for the seasonal change of soil microbial community in the wetland of the coal mining subsidence area of Jining.

**Supplementary Information:**

The online version contains supplementary material available at 10.1186/s13568-023-01653-5.

## Introduction

Wetlands, as one of the world’s three major ecosystems, not only play an important role in supporting the formation of soil, supplying food and fresh water, and regulating the climate, but are also important habitats for aquatic plants and animals, and important bases for material cycling and biochemical reactions (Shen et al., 2019; Camacho-Valdez et al. 2014). Coal mining subsidence leads to a drop in the water table and subsidence of the collapsed surface to form a waterlogged area, causing significant environmental changes and destruction of the original ecosystems, resulting in the formation of a unique nascent wetland in the coal mining subsidence area (Chang Jiang et al., 2017; Zhou et al. 2014). As a special type of wetland, new wetland in coal mining subsidence area is obviously different from natural wetland and other types of constructed wetland in terms of formation process and human disturbance degree. Studies on coal mining subsidence land at home and abroad are more focused on subsidence land reclamation technology, subsidence land ecological restoration technology, and subsidence land restoration and management technology (Li et al. 2022; Rouhani et al. 2023; Bi et al. 2018). There are relatively few studies on the evolution law of nascent wetland ecosystems in coal mining subsidence areas, which focus on the change of soil fertility, biochemical properties of soil, and microbial communities in subsidence areas (Ma et al. 2019; Sun et al. 2023; Fang et al. 2023).

Soil nutrients and physicochemical factors are important parameters for evaluating the evolution of soil ecosystems in wetland. Land subsidence caused by coal mining has resulted in significant changes in environmental factors such as soil nitrogen, phosphorus and organic matter content. A study carried out by Ma et al. (2019) showed that soil water content (SWC), total nitrogen (TN), dissolved organic carbon (DOC), ammonia nitrogen (NH_4_^+^-N), nitrate nitrogen (NO_3_^−^-N), available phosphorus (AP), and available potassium (AK) were significantly lower in the subsidence zone than in the control zone, and that ground subsidence had a significant effect on the characteristics of the soil, such as SWC, nutrients, enzyme activity, and bacterial population (Ma et al. 2019). Another study on coal mining subsidence areas in central China found that the chemical and biological properties of soils showed strong seasonal correlations, with the highest soil TC and AP in the spring, the highest TN in the fall, and the highest AN in the summer (Sun et al. 2023).

Soil microorganisms are the decomposers of wetland ecosystems, and they play an important role in material circulation and energy flow. On the one hand, soil microorganisms make significant contribution to the transfer and conversion of N and P elements in soil. For example, soil microbes can directly affect the content of metal oxides in the soil, promote the formation of amino sugars, and ultimately promote the accumulation of soil organic carbon (Xu et al. 2023), and they can also convert immobilized nitrogen in the environment into microbial biomass nitrogen, which can then be mineralized or converted into stable soil organic matter, thus realizing the translocation and transformation of soil nitrogen (Li et al. 2021). On the other hand, microbial diversity is directly affected by the content and presence patterns of nutrients such as nitrogen and phosphorus. For example, the application of compound fertilizer significantly increased the nitrogen and phosphorus content of coal mining collapse soil, increased soil bacterial diversity, and improved bacterial community structure (Meng et al. 2023). Adding biochar can similarly alter the diversity of soil bacteria and fungi as well as community structure (Liu et al. 2019). Therefore, soil microbial diversity plays an important role in reflecting the condition of soil fertility and the evolution of wetland ecosystem. It has become an important indicator of wetland evaluation system (Gao et al. 2021).

In recent years, the studies of soil microbial community structure in wetlands of coal mining subsidence areas have been gradually carried out. Li Jinlan used phosphor lipid fatty acid (PLFA) analysis to research the microbial community structure of reclaimed soil from the coal mining subsidence area of Xiangyuan Wuyang coal mine in Changzhi, Shanxi. They found that the increase in total soil microbial PLFA, bacterial PLFA and fungal PLFA was more pronounced and the community structure of soil microbes was more changed under chemical fertilizer and organic fertilizer treatments (Li et al. 2010). Li et al. examined the diversity of bacteria and soil physicochemical properties of reclaimed sites and neighboring coal mining subsidence areas using 16 S rDNA pyrophosphate sequencing. The results showed that the reclaimed area had richer bacterial diversity and higher soil organic matter (SOM) and total nitrogen (TN) content (Li et al. 2014). Shi et al. studied the coal mining subsidence area in the windswept region of western China and found that the main factors driving the structural changes of the bacterial community were electrical conductivity (EC), water content (WC), and soil depth, which revealed the effects of ground subsidence on the soil bacterial community and the response of soil bacterial community to changes in the soil environment (Shi et al. 2017). In addition, a large number of scholars have devoted themselves to the study of the effects of the arbuscular mycorrhizal fungi (AMF) on coal mining subsidence areas. Bi et al. investigated the effects of different inoculation treatments on AMF fungal communities and soil remediation sustainability in coal mining subsidence areas, pointing out that the diversity and structure of AMF fungal communities are sensitive indicators for monitoring environmental changes in subsidence areas (Bi et al. 2018). Studies by Li et al. have shown that arbuscular mycorrhizal fungi are associated with the potential activity of nitrogen-harvesting enzymes in soils and with influencing nitrogen cycling in sedimentary soils from coal mines (Xiao et al., 2019). Qiu et al. found that AMF inoculation was an effective method to improve soil fertility and restore vegetation communities under harsh conditions such as drought (Qiu et al. 2019). Recently, Gao et al. investigated the effect of coal mine subsidence on the distribution of microbiota and their functional genes in farmland with the help of 16 S rRNA gene sequencing technology. They found that a core microbial community existed in the coal mining subsidence area, and the coal mine subsidence altered the distribution of microbial community and their functional genes in farmland, and proposed that the concentrations of soil moisture, pH, NH_4_^+^, and Ca^2+^ were the main factors affecting the distribution of microbial community and their functional genes (Gao et al. 2023). Fang et al. explored the diversity and ecological assembly process of aquatic bacterial communities in lakes during the subsidence period in winter and summer, and found that seasonal changes had a significant effect on the structure and diversity of bacterial communities in subsiding lakes. Moreover, the α-diversity and functional diversity of bacterial communities were higher in summer than in winter, and temperature and chlorophyll a were the most important influences on the seasonal changes of microbial communities (Fang et al. 2023). With the deepening of research, the study of soil microbes in nascent wetlands in coal mining subsidence areas has received more and more extensive attention. However, there are fewer studies on the seasonal variations of soil microbial diversity in coal mining subsidence areas and their associations with soil physicochemical properties, which still need to be supplemented.

16 S rRNA amplicon sequencing technology, which uses universal primers designed for conserved regions for PCR amplification, sequencing high-variation regions and strain identification, has become an important means to study the composition and structure of microbial communities in environmental samples. (Caporaso et al., 2011; Gao et al. 2023). Therefore, in this study, we intend to investigate the seasonal changes of soil microbial composition and functional diversity in the nascent wetland of coal mining subsidence area with the help of 16 S rRNA gene sequencing technology. Meanwhile, the seasonal changes of nutrient elements and physicochemical properties of the soil in the coal mining subsidence area were measured, so as to analyze the correlation between the microbial diversity of the soil and the changes of nutrient elements and physicochemical properties of the soil, to deepen the understanding of the ecological evolution process of the soil in the wetland in the coal mining subsidence area. The study will provide scientific data for the scientific development of ecological management and restoration measures for coal mining wetlands, and it will further investigate the ecological value and environmental development mechanism of coal mining wetlands.

## Materials and methods

### Soil sample collection

In this study, soil samples were collected from Xinglong Wetland (35°27′6″N,116°51′31″E) and Dongtan Coal Mine Coal Mining Subsidence Area (35°31′45″N,116°51′53″E) in Jining, Shandong Province, China. The habitat type of each sampling site was detected, and the wet area near the water was selected as the sampling site. Four sampling sites were selected for each sample plot, and three soil samples were collected from each site and blended into one soil sample, for a total of 16 soil samples collected in both seasons. When collecting soil at the sampling point, the soil surface impurities should be removed first to make the ground bare. If there are plants at the sampling point, the rhizosphere soil should be further removed. Then 10 ml soil samples were collected with aseptic tubes for subsequent soil microbial determination and analysis. The sample should be sealed immediately after collection, and the sample information should be recorded and put into the sampling box. After returning to the laboratory, the collected samples were immediately sent for sequencing. In addition, 10 kg of soil samples were collected at the same sampling point using a sampler, and the information was recorded and then sealed for the determination of environmental factors.

### Detection of soil environmental factors and soil nutrient elements

For environmental factors, we determined the temperature, pondus hydrogenii (pH), dry matter content (DMC), moisture content (MC), total phosphorus (TP), available phosphorus (AP), total nitrogen (TN) and total organic carbon (TOC) values of soil samples, and then the results were calculated according to Table (Table S1). For data accuracy, soil temperature was measured in real-time during sampling. The remaining indicators were tested after treating the soil samples in the laboratory. The soil samples were naturally dried indoors, ground after breaking up the clods, and sieved for 100 mesh for subsequent determination. The pH values were determined using the NY/T 1377–2007 determinometer, and the results were accurate to two decimal places. Soil samples were treated by microwave heating to obtain the dry matter content. The MC value was determined by NY/T 52-1987 using the weight difference method. Total phosphorus was determined by NY/T 88-1998, and available phosphorus was determined by HJ 704–2014. The results were accurate to the last two decimal places. The contents of total nitrogen and total organic carbon were determined by using multi N/C 3100 analyzer with reference to Kirschler sodium benzoate method, combustion oxidation method and non-dispersive infrared method (Wang et al. 2020; Sui et al. 2021; Shang et al. 2020; Chen et al., 2023). All the experimental processes were completed in the Experimental Center of the College of Life Science, Qufu Normal University.

### Soil microbial genomic DNA extraction, PCR amplification, library construction and sequencing

Genomic DNA was extracted from soil samples by CTAB method. Upon completion of the extraction, the DNA was examined with 1% agarose gel electrophoresis, and an appropriate amount of sample DNA was taken in a centrifuge tube and sterile water was injected to dilute the samples to 1 ng/µL. The V4 region of the 16 S rRNA gene was amplified using primers 515 F-806R. All PCR reaction systems were supplemented with 15 µL of Phusion® High-Fidelity PCR Master Mix (New England Biolabs), 0.2 µM primers, and 10 ng of genomic DNA template. Thermal cycling was as follows: initial denaturation at 98 °C for 1 min, denaturation at 98 °C for 10 s, annealing at 50 °C for 30 s, and extension at 72 °C for 30 s. After 30 cycles, incubation was performed at 72 °C for 5 min. PCR products were detected by electrophoresis using agarose gel with 2% concentration. The PCR products that passed the test were purified by magnetic beads, quantified by enzyme labeling, and mixed in equal amounts according to the concentration of PCR products, and the PCR products were detected by electrophoresis using a 2% agarose gel after sufficient mixing. The products were recovered using a universal DNA purification and recovery kit (TianGen) for the target bands. Library construction was performed using NEB Next® Ultra™ II FS DNA PCR-free Library Prep Kit (New England Biolabs), and the constructed library was quantified by Qubit and Q-PCR. After the library was qualified, the PE250 was up-sequenced using Illumina NovaSeq 6000 to generate 250 bp pairs of sequences.

### Data quality control

Break down the data, separate the data from each sample, and deal with the data of each sample through splicing, quality filtering, and removal of the chimeric sequence, so as to obtain the original label, the clean label and the valid tag. The analysis was done with FLASH 1.2.11.

### Analysis of alpha diversity and beta diversity of soil microbiota

The Uparse algorithm (Uparse v7.0.1001) (Edgar RC et al., 2013) was used to cluster all Effective Tags from all samples with 97% concordance for OTUs clustering (Operational Taxonomic Units), and to filter representative OTUs sequences for subsequent species annotation.

The alpha indices of Observed-otus, Chao1, Shannon, Simpson, ace, Goods-coverage, PD whole tree, etc., were calculated using Qiime software (Version 1.9.1). Statistical tests were performed using R software to evaluate the differences between the alpha diversity indices of the different groups.

Beta diversity analysis was performed on Qiime (Version 1.9.1) software, and Anosim analysis was used to test the reasonableness of the groupings. Differences in annotated species abundance between taxa at different taxonomic levels were calculated by the MetaStat test. In order to reflect between-group and within-group differences in the sample, principal component analysis (PCA), principal co-ordinates analysis (PCoA), and non-metric multi-dimensional scaling (NMDS) were performed. LDA effect size (LEfSe) software was used to detect differences in abundance across taxonomic classes (phylum, class, order, family, and genus) between groups and to find biomarkers that were key contributors to differences between groups.

### Functional annotation of soil microbial genes

Tax4Fun functional prediction was performed on the sequencing data to analyze the functional composition of the microbial community in the samples. The whole genome 16 S rRNA gene sequence of prokaryotes was extracted from KEGG database and compared to SILVA SSU Ref NR database by BLASTN algorithm to establish correlation matrix. The functional information of the KEGG database annotated by UProC and PAUDA was corresponding to the SILVA database to obtain the functional annotation of the SILVA database.

### Analysis of the relationship between environmental factors and microbial communities

The Spearman’s Correlation Analysis (Algina, J et al., 1999) was used to study the variation of environmental factors and the richness of microbial species (alpha diversity). CCA analysis (Sheik et al. 2012) is a reflection of the relationship between the microbiota and the environment, and the significance of the environmental factors was determined by CCA analysis using the envfit function to determine the major environmental factors influencing the sample distribution.

## Results

### Soil environmental factors in coal mining subsidence area

The results of soil environmental factors showed no significant differences in pH, moisture content (MC) and total nitrogen (TN) values between the summer and winter groups. Temperature (Temp), total phosphorus (TP) and available phosphorus (AP) were significantly higher (*p* < 0.05) in summer soil samples than in winter, whereas total organic carbon (TOC) as well as dry matter content (DMC) were significantly higher (*p* < 0.05) in soil samples in winter than in summer (Supplementary Table 1).

### Species annotations and metastat tests

The 16 soil samples were categorized into winter (WI) and summer (SU) according to the sampling season, and a total of 16,050 OTUs were obtained after sample gene noise reduction. All OTUs were annotated to 68 bacterial phyla, 708 bacterial families, 1,256 bacterial genera, and a large number of unclassified species. Venn diagram analysis showed a total of 4,074 OTUs in two groups. There were 8217 OTUs specific to the group SU and 7833 OTUs specific to the group WI. The number of OTUs was slightly higher in the group SU than in the group WI (Supplementary Fig. 1).

**Fig. 1:**
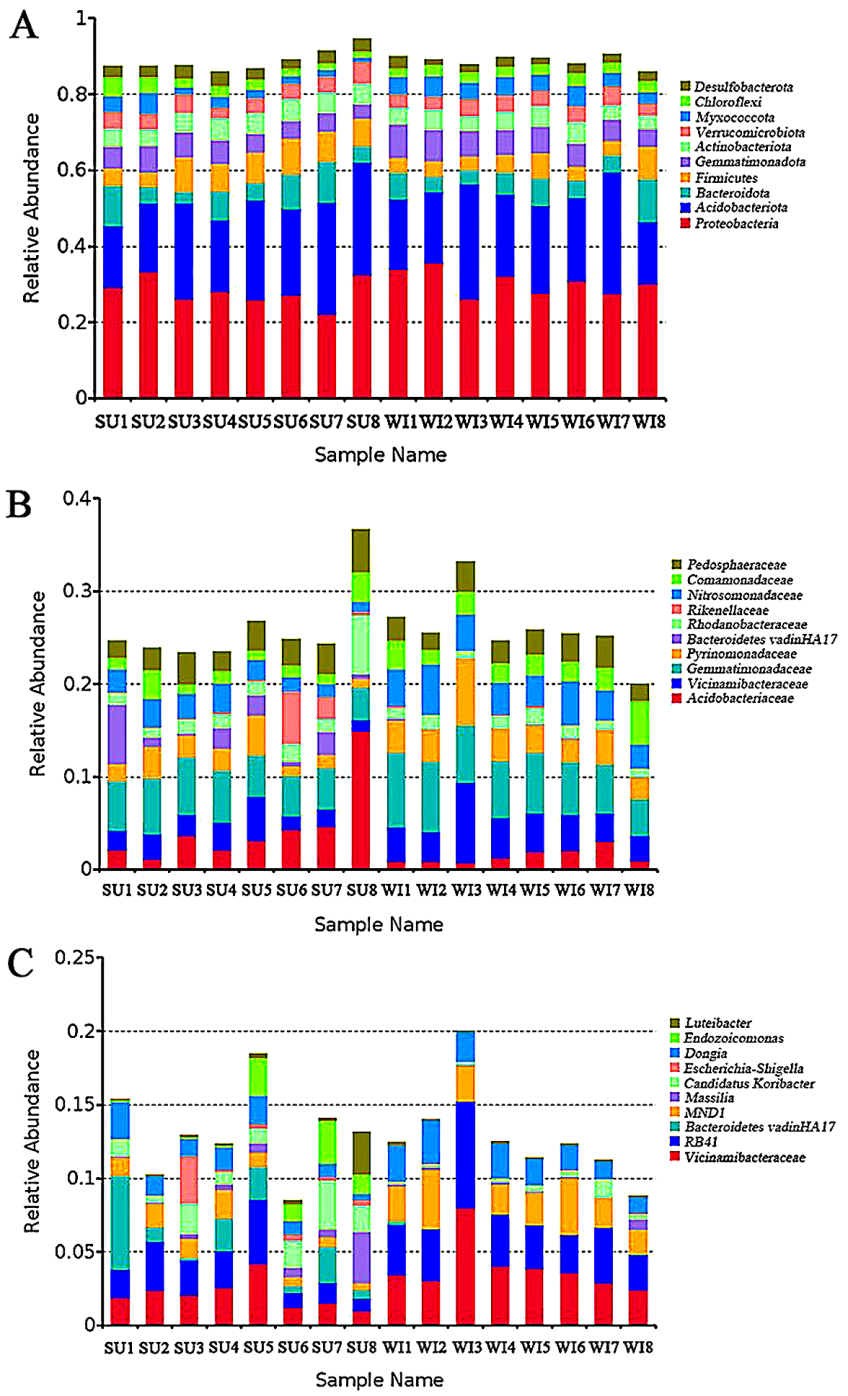
Relative abundance of annotated genes at phylum, family and genus levels. The top 10 phylum **(A)** family **(B)** and genus **(C)** with the highest abundance in each group were selected

At the level of phylum classification, the phylum with the highest abundance of soil microorganisms in all samples in both seasons were *Proteobacteria* (29.30%), *Acidobacteriota* (23.00%), *Bacteroidota* (6.40%), *Firmicutes* (6.09%), *Gemmatimonadota* (6.07%), *Actinobacteriota* (4.98%), *Verrucomicrobiota* (4.18%), *Myxococcota* (3.47%), *Chloroflexi* (2.79%) and *Desulfobacterota* (2.56%) (Fig. 1A). The total abundance of *Proteobacteria* and *Acidobacteriota* accounts for about half of all annotated phyla (52.30%). At the family level, the most abundant families were *Gemmatimonadaceae* (5.60%), *Vicinamibacteraceae* (3.31%), *Acidobacteriaceae* (2.98%), *Nitrosomonadaceae* (2.98%), *Pyrinomonadaceae* (2.95%), *Pedosphaeraceae* (2.93%), *Comamonadaceae* (2.14%), *Rhodanobacteraceae* (1.66%), *Bacteroidetes vadinHA17* (0.99%), *Rikenellaceae* (0.61%) (Fig. 1B). At the genus level, *Vicinamibacteraceae* (3.01%), *RB41* (2.95%), *MND1* (1.99%), *Dongia* (1.63%), *Candidatus Koribacter* (1.01%), *Bacteroidetes vadinHA17* (0.99%), *Endozoicomonas* (0.52%), *Massilia* (0.46%), *Escherichia-Shigella* (0.30%) and *Luteibacter* (0.27%) were the genera with high abundance (Fig. 1C).

Metastat tests of intergroup variability showed that at the phylum level, the abundance of *Firmicutes* and *Desulfobacterota* was significantly higher in the group SU than in the group WI, and the abundance of *Myxococcota* was significantly higher in the group WI than in the group SU (Supplementary Fig. 2). At the family level, the abundance of *Vicinamibacteraceae*, *Nitrosomonadaceae*, and *Pyrinomonadaceae* was significantly higher in the group WI than in the group SU (*p* < 0.05). The abundance of *Bacteroidetes vadinHA17*, *Acidobacteriaceae*, and *Rikenellaceae* had significantly higher abundance group SU than group WI (*p* < 0.05) (Supplementary Fig. 3). At the genus level, the top ten genera in abundance were significantly different between the two groups, except *Massilia*, *Dongia* and *Luteibacter*.

### Alpha diversity analysis

In order to compare the diversity of soil microbial communities in the subsidence area, we calculated the Alpha diversity indices of different samples at the 97% consistency threshold and performed statistical tests on each index. The results showed that the indices were not significantly different between the two groups (Table 1).

**Table 1:** Alpha Diversity Indices and t-test *p*-value of soil microorganisms in summer and winter subsidence areas

Group	Chao1	Shannon	Simpson	Observed OTUs	Goods coverage
SU	3006.026	9.943	0.996	2626.389	0.996
WI	2720.779	10.475	0.998	3130.111	0.994
*p*-value	0.519	0.078	0.086	0.484	0.954

### Analysis of microbial community differences in summer and winter subsidence areas

Anosim results based on the Bray Curtis algorithm showed that intergroup differences were greater than intragroup differences (R > 0 and *p* < 0.05), indicating significant seasonal differences in soil microbial diversity in the subsidence area (Table 2).

**Table 2:** Anosim analysis based on Bray Curtis distance

Group	R	*P*
SU-WI	0.661	0.005

**Fig. 2:**
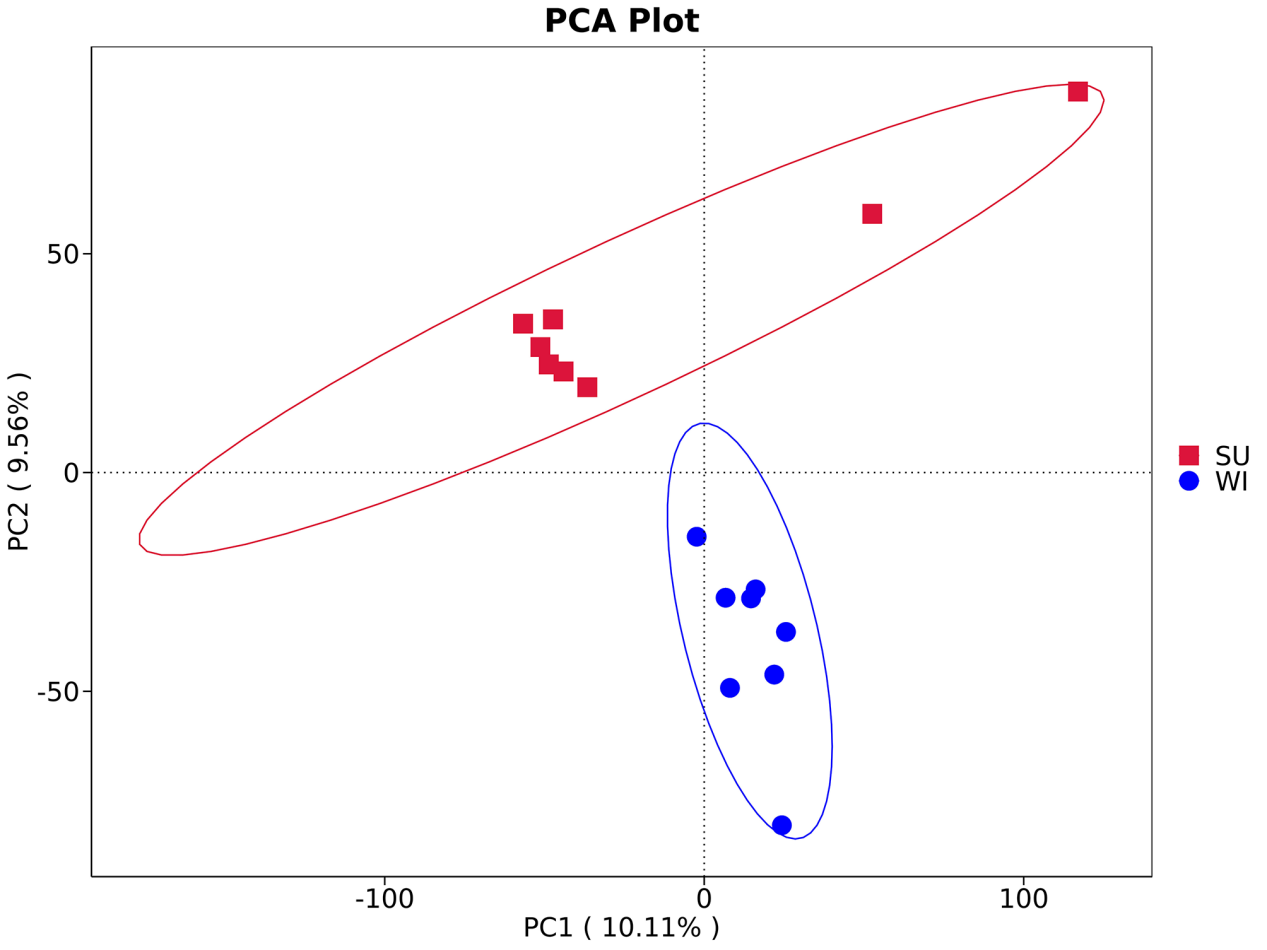
PCA analysis based on the abundance of annotated genes showed that samples from the groups SU and WI clustered individually

Beta diversity analysis explores similarities or differences in community composition between subgroups of samples by analyzing species diversity between groups of different habitats or microbial communities in a comparative manner. The results of PCA analysis showed that the samples in the group SU were clearly clustered together, and the samples in the group WI were also clustered together. A clear divergence was presented between the two groups (Fig. 2). The results of NMDS and PCOA analyses also supported the aggregation of each sample separately by season (Supplementary Figs. 4–5), suggesting that soil microbial diversity in coal mining subsidence area is correlated with the seasons.

**Fig. 3:**
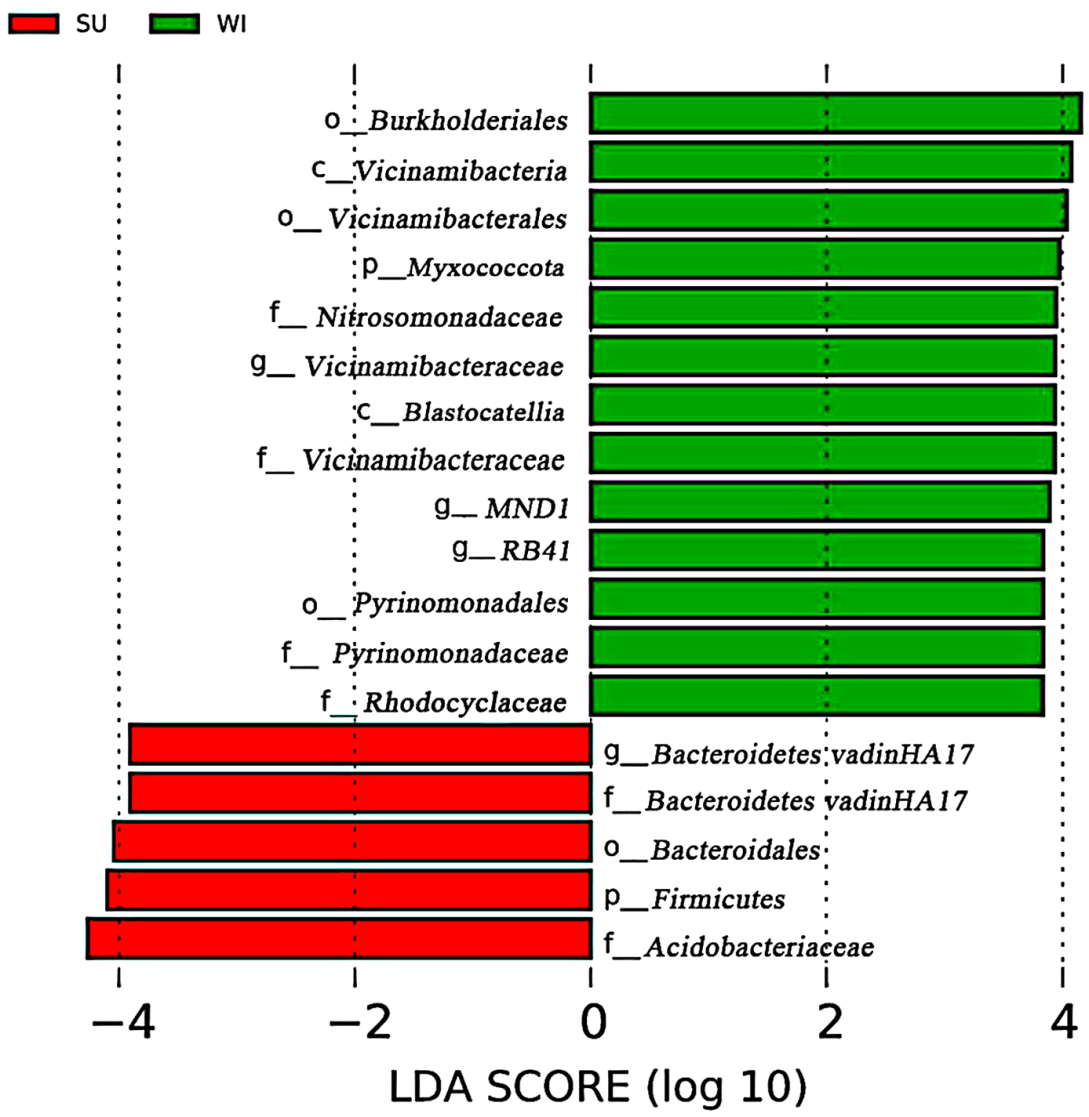
LEfSe analysis showed respective biomarkers in groups SU and WI

After LEfSe analysis, Biomarkers with statistically significant differences between groups were sought. LEfSe analyses showed that the differences in soil bacteria in the subsidence area between the two seasons were mainly in *Firmicutes* and *Myxococcota*. The families that were statistically different between the two groups were *Nitrosomonadaceae*, *Vicinamibacteraceae*, *Pyrinomonadaceae*, *Rhodocyclaceae*, *Bacteroidetes vadinHA17* and *Acidobacteriaceae*. Statistically different genera were *Vicinamibacteraceae*, *MND1*, *RB41* and *Bacteroidetes vadinHA17* (Fig. 3).

### Gene functional annotation and functional diversity analysis

In database, microbial genes in the two-season subsidence area were mainly enriched in the Metabolism (47.71%), Genetic Information Processing (20.68%), and Environmental Information Processing (13.51%) metabolic pathways. Among them, Carbohydrate metabolism, Amino acid metabolism, Energy metabolism, Lipid metabolism pathway, Metabolism of cofactors and vitamins, Nucleotide metabolism in Metabolism Processing, Membrane transport, Replication and repair pathway during Genetic Information Processing, Translation and Signal transduction during Environmental Information Processing had the highest number of genes enriched (Supplementary Fig. 6).

**Fig. 4:**
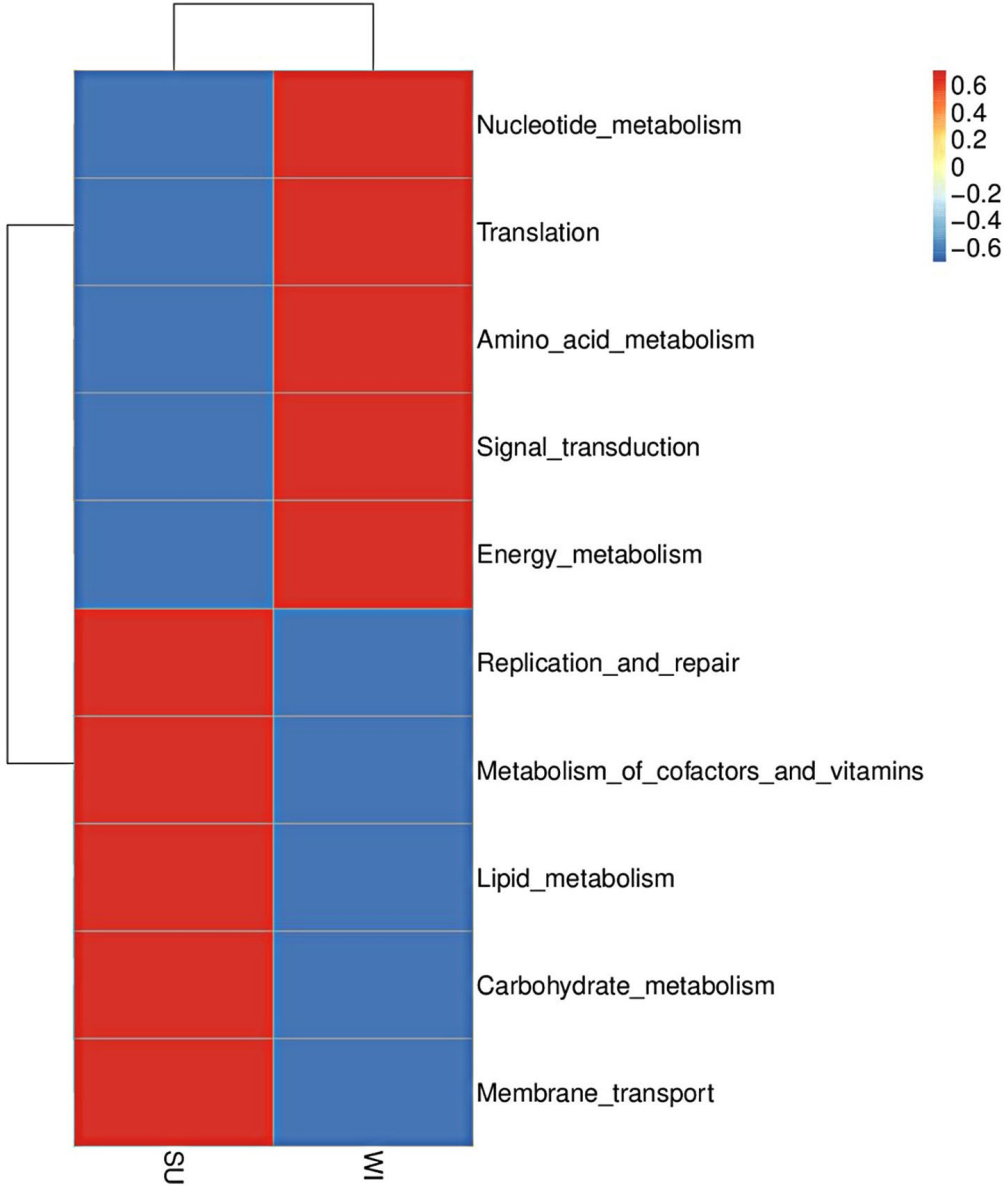
Comparison of annotation function abundance between groups SU and WI based on KEGG database

Comparison of annotation function abundance between groups SU and WI revealed that in database, group SU had higher abundance in Membrane transport and Replication and repair during Genetic Information Processing, Carbohydrate metabolism, Lipid metabolism pathway, Metabolism of cofactors and vitamins during Metabolism. The group WI was more enriched in Nucleotide metabolism, Amino acid metabolism, Energy metabolism during Metabolism Processing, Translation and Signal transduction during Environmental Information Processing. (Fig. 4).

### Correlation analysis between soil environmental factors and microbial diversity

**Fig. 5:**
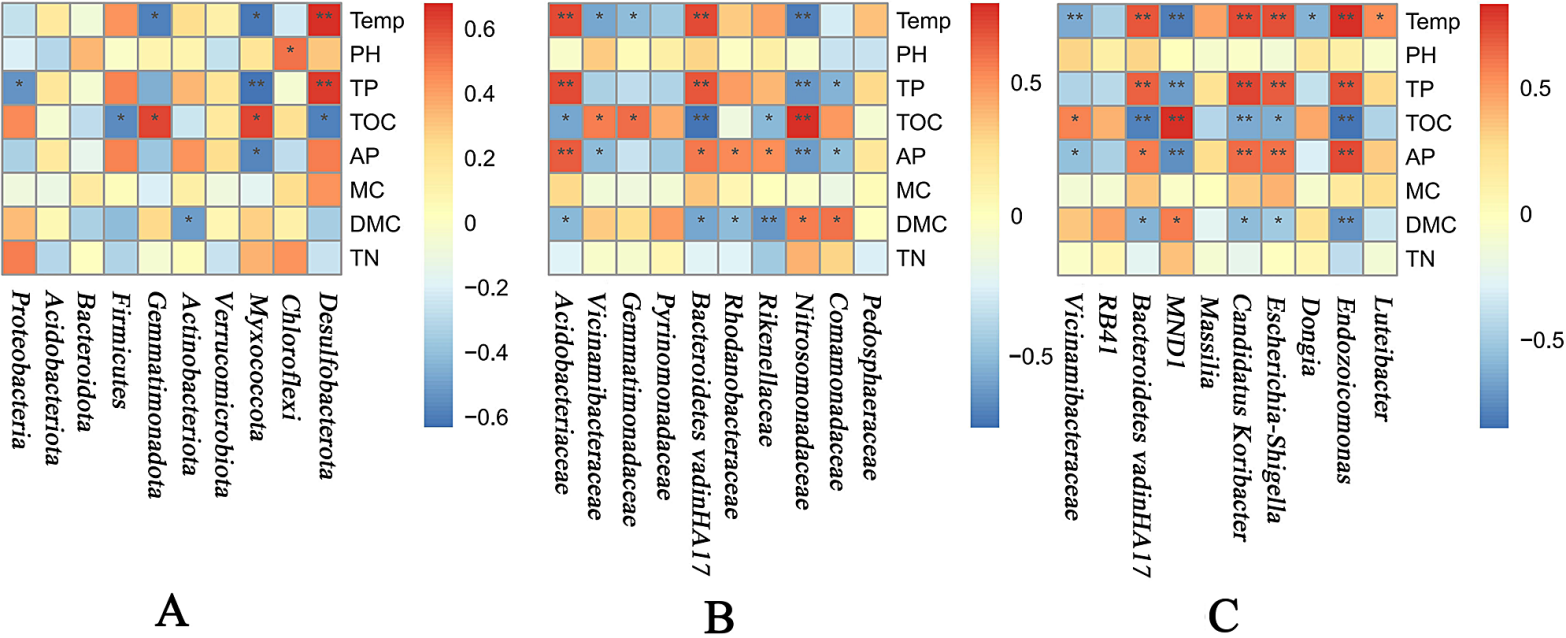
Results of Spearman correlation analysis at phylum(A), family(B) and genus(C) level

Spearman correlation analysis showed that the abundance of *Desulfobacterota* was highly significantly positively correlated with both Temp and TP (*p* < 0.01), *Gemmatimonadota* and *Myxococcota* were significantly positively correlated with TOC (*p* < 0.05), *Chloroflexi* was significantly positively correlated with pH (*p* < 0.05), *Myxococcota* was significantly negatively correlated with AP (*p* < 0.05), and *Gemmatimonadota* was significantly negatively correlated with DMC (*p* < 0.05). The abundance of *Firmicutes* and *Desulfobacterota* was significantly negatively correlated with TOC (*p* < 0.05). *Gemmatimonadota* and *Myxococcota* were significantly negatively correlated with Temp (*p* < 0.05), and *Myxococcota* and *Proteobacteria* were significantly negative correlation with TP (*p* < 0.05). At the family level, *Acidobacteriaceae*, *Bacteroidetes vadinHA17* showed highly significant positive correlation (*p* < 0.01) with both Temp and TP, and *Vicinamibacteraceae*, *Gemmatimonadaceae*, *Nitrosomonadaceae* showed significant positive correlation (*p* < 0.05) with the TOC showed significant positive correlation (*p* < 0.05). *Vicinamibacteraceae*, *Gemmatimonadaceae*, and *Nitrosomonadaceae* showed significant negative correlation with Temp (*p* < 0.05), and *Nitrosomonadaceae*, *Comamonadaceae* showed significant negative correlation with TP (*p* < 0.05). *Acidobacteriaceae*, *Bacteroidetes vadinHA17*, and *Rikenellaceae* were significantly negatively correlated with TOC (*p* < 0.05). *Acidobacteriaceae*, *Bacteroidetes vadinHA17*, *Rhodanobacteraceae*, *Rikenellaceae* showed significant positive correlation (*p* < 0.05) with AP and significant negative correlation (*p* < 0.05) with DMC, while *Nitrosomonadaceae*, *Comamonadaceae* showed significant negative correlation (*p* < 0.05) with AP and significant positive correlation (*p* < 0.05) with DMC. At the genus level, *Bacteroidetes vadinHA17*, *Candidatus Koribacte*r, *Escherichia-Shigella*, *Endozoicomona*s, and *Luteibacter* were significantly and positively correlated (*p* < 0.05) with Temp, while *Bacteroidetes vadinHA17*, *Candidatus Koribacter*, *Escherichia-Shigella*, *Endozoicomonas* showed highly significant positive correlation (*p* < 0.01) with TP. *Vicinamibacteraceae*, *MND1*, and *Dongia* were significantly negatively correlated with Temp (*p* < 0.05), and *MND1* was significantly negatively correlated with TP (*p* < 0.05). *Vicinamibacteracea*e, *MND1* was significantly positively correlated with TOC (*p* < 0.05) and negatively correlated with AP (*p* < 0.05). *MND1* was significantly positively correlated with DMC (*p* < 0.05) *Bacteroidetes vadinHA17*, *Candidatus Koribacter*, *Escherichia-Shigella*, and *Endozoicomonas* showed a significant positive correlation (*p* < 0.05) with AP, but a significant negative correlation (*p* < 0.05) with TOC and DMC (Fig. 5).

**Fig. 6:**
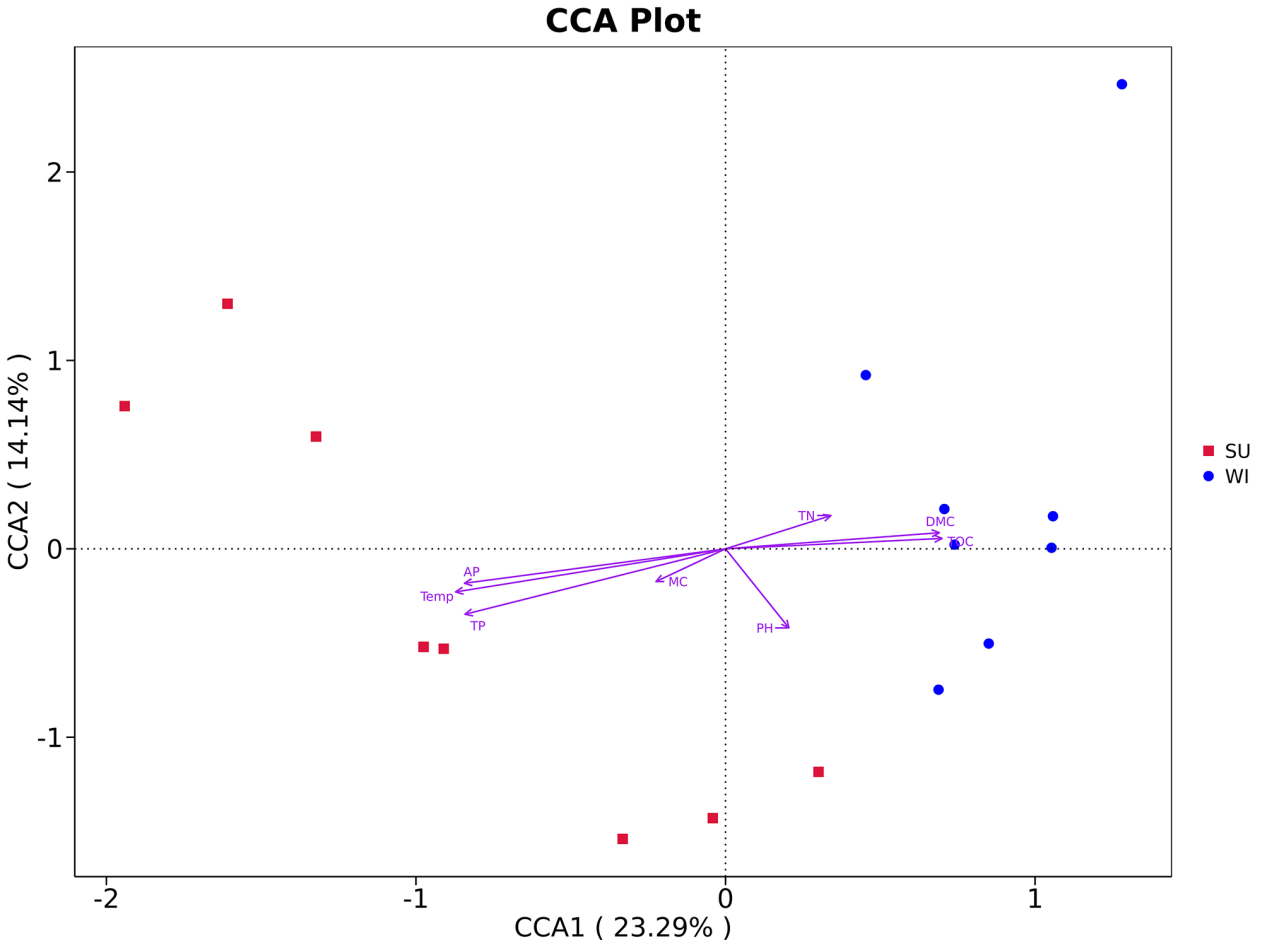
The results of CCA analysis showed the influence of environmental factors on the abundance of soil bacterial OTU

The results of CCA analysis indicated that the samples collected in summer were concentrated in the second and third quadrants, while those in the winter were concentrated in the first and fourth quadrants. The correlation of environmental factors with CCA1, CCA2 was 23. 29% and 14. 14%, respectively. Temp, AP and TP contents (*p* < 0.01) were significantly correlated with the abundance of soil microorganisms in the summer subsidence area, while DMC and TOC contents (*p* < 0.01) were significantly correlated with the abundance of soil microorganisms in the winter subsidence area (Fig. 6).

## Discussion

Soil microbes play an important role in maintaining the ecological balance of wetlands in coal mining subsidence areas. Studies have shown that there is a core colony of soil microorganisms in coal mining subsidence areas, which determines the community composition of microorganisms in the whole subsidence area (Gao et al. 2023). The microorganisms with the highest abundance in the soil of the Jining coal-mining subsidence wetland were *Proteobacteria* and *Acidobacteriota*, and the combined abundance of the two phyla accounted for about half (52.3%) of all annotated phyla. The top ten clades in summer abundance were *Proteobacteria*, *Acidobacteriota*, *Firmicutes*, *Bacteroidota*, *Gemmatimonadota*, *Actinobacteriota*, *Verrucomicrobiota*, *Desulfobacterota*, *Chloroflexi* and *Myxococcota*, while the top ten clades in winter abundance were *Proteobacteria*, *Acidobacteriota*, *Gemmatimonadota*, *Bacteroidota*, *Firmicutes*, *Acidobacteriota*, *Myxococcota*, and *Verrucomicrobiota*, *Chloroflexi* and *Desulfobacterota*. This result is similar to other studies of soil microbes in coal mining subsidence areas (Meng et al. 2023; Fang et al. 2023). In addition, in our study, although alpha diversity analysis showed no significant difference in soil microbes between the two seasons, the summer soil microbial community annotated more OTUs and the number of OTUs specific to the two seasons far exceeded the number of shared OTUs (Supplementary Fig. 1). It indicated that there was respective dominant microbiota in the soil of the wetland of the subsidence area in winter and summer to adapt to the challenges brought by seasonal changes and to maintain the ecological balance of the wetland of the coal mining collapsed area.

*Proteobacteria* was the most abundant phylum of soil microbes in the wetland of Jining coal mining subsidence area. *Proteobacteria* are known to be common bacteria in soil, and there are many methanophilic bacteria in this phylum. In wetland ecosystems, these methane-oxidizing bacteria can consume large amounts of CH_4_ produced by the deep anoxic layer and maintain wetland nitrogen balance (Siljanen et al., 2012). They can also degrade organic matter and effectively reduce sediment pollution (Shen et al. 2020). In our study, *Proteobacteria* were significantly and negatively correlated with soil TP content, however *Proteobacteria* abundance did not differ significantly between seasons. Metastat intergroup variability tests showed that the abundance of the *Nitrosomonadaceae* (family *Proteobacteria*) was significantly higher in winter than in summer. *Nitrosomonadaceae* were associated with the nitrogen cycle and were an important component of soil nitrite bacteria (Cua and Stein et al., 2011), and were positively correlated with soil CO_2_ emissions, which significantly improved soil respiration (Wang et al. 2022), and also promoted soil carbon mineralization (Li et al., 2020). In our study, *Nitrosomonadaceae* was positively correlated with soil TOC and DMC content. It has been found that *Nitrosomonadaceae* dominate in low-temperature freshwater environments (Cébron et al. 2003). This was supported by our study, in which *Nitrosomonadaceae* abundance was higher in winter and the abundance of *Nitrosomonadaceae* was significantly negatively correlated with temperature. Therefore, we hypothesize that the enrichment of *Nitrosomonadaceae* in winter improves soil respiration in wetlands of coal mining subsidence areas, promotes CO_2_ emission, and participates in maintaining the balance of soil carbon and nitrogen cycles.

*Acidobacteriota* was considered one of the major phyla of soil microbes with a wide range of temperature tolerance and was found in both cold or warm regions (Conradie et al., 2021). They can participate in the degradation of polymers such as chitin, cellulose, hemicellulose and xylan (Belova et al. 2018). In addition, it has been shown that *Acidobacteriota*, which was partially anaerobic in wetland systems, was able to utilize both inorganic and organic sulfur molecules to participate in sulfur metabolism to conserve energy (Hausmann et al. 2018). In our study, the results of Spearman’s correlation analysis showed that *Acidobacteriota* was significantly and positively correlated with Temp, TP and AP contents of wetland soils in the coal mining subsidence area of Jining. Shelyakin’s study also found a higher proportion of *Acidobacteriota* in environments rich in organic matter and effective phosphorus (Shelyakin et al. 2022), hypothesizing that the amount of phosphorus in the environment is one of the factors influencing the distribution of *Acidobacteriota*. The abundance of *Bacteroidota* in the wetland soil of Jining coal mine subsidence area was second only to *Proteobacteria* and *Acidobacteriota*, and the results of Metastat intergroup variability test showed that the abundance of *Bacteroidota* was significantly higher in summer than in winter. It has been found that *Bacteroidota* can obtain organic matter from algae, so their abundance and distribution were influenced by the algae in the environment (Shen et al. 2020). We hypothesize that algal blooms in wetlands occur during the summer months due to favorable temperatures and environments, leading to a large enrichment of *Bacteroidota* bacteria that utilize algae for organic matter.

Seasonal variations in wetland soil microbes are due to different soil physicochemical properties, such as temperature, enzyme activity, content and form of carbon, nitrogen, phosphorus and other elements present in the soil and soil pH. Siljanen et al. found that seasonally varying hydrologic and temperature conditions in eutrophic northern coastal wetlands affect the community composition and functional diversity of methanotrophic bacteria (Siljanen et al. 2012). Zhang et al. revealed the seasonal changes of bacterial communities in the wetland of Shuangtaizi estuary paddy field by high-throughput sequencing technology, and found that the soil properties changed significantly with the seasons, and pH, C, and TN were the key factors constituting the seasonal changes of microbial communities (Zhang et al. 2020). Zhang et al. found a close association between microbial biomass and soil nutrients such as TP, AP, TK and AK (Zhang et al. 2021). Relatively few studies have been conducted on the changes in soil microbial diversity in coal mining subsidence areas and their association with soil physicochemical properties. The study by Fang et al. indicated that changes in bacterial community structure were related to five environmental factors: temperature, chlorophyll a, TN, NO_3_^−^ and NO_2_^−^ and that temperature and chlorophyll a were the most important influences on seasonal variations of microbial communities in coal-mining settlement lakes (Fang et al. 2023). Sun et al. indicated that soil TC and AP contents were highest in spring, soil TN in fall, and soil AN in summer. Soil chemical and biological properties showed strong spatial autocorrelation in all seasons (Sun et al. 2023). Gao et al. found that soil moisture, pH, NH_4_^+^ and Ca^2+^ concentrations were the main factors affecting soil microbial communities and their functional gene distributions in the coal mining subsidence area (Gao et al. 2023). In our study, temperature, TP, AP, TOC and DMC were the key factors in the seasonal variation of soil microbial communities in the wetland of the coal mining subsidence area in Jining. The results of CCA analysis showed that Temp, AP and TP contents in the summer subsidence area were significantly correlated with the abundance of soil microorganisms, and that the dominant bacteria *Acidobacteriaceae*, *Bacteroidetes vadinHA17* were significantly positively correlated with Temp, AP and TP, and *Desulfobacterota* was significantly positively correlated with Temp and TP. The contents of DMC and TOC in the winter collapse zone were significantly correlated with the abundance of soil microorganisms. The dominant bacteria *Myxococcota*, *Vicinamibacteraceae*, *Gemmatimonadaceae*, and *Nitrosomonadaceae* showed significant positive correlation with TOC, while *Nitrosomonadaceae* and *Comamonadaceae* showed significant positive correlation with DMC. In addition, although pH and TN have been shown to be important factors controlling microbial community aggregation (Bahram et al. 2018; Zhang et al. 2020), there were no significant changes in soil pH and TN content between the two seasons in our study.

### Electronic supplementary material

Below is the link to the electronic supplementary material.

**Supplementary Material 1**: Venn diagram shows number of genes common and unique to the groups SU and WI

**Supplementary Material 2**: Results of Metastat test at the phylum level. * means significant difference (p < 0.05), ** means extremely significant difference (p < 0.01)

**Supplementary Material 3**: Results of Metastat test at the family level. * means significant difference (p < 0.05), ** means extremely significant difference (p < 0.01)

**Supplementary Material 4**: PCOA analysis based on the abundance of annotated genes showed that samples from the groups SU and WI clustered individually

**Supplementary Material 5**: NMDS analysis based on the abundance of annotated genes showed that samples from the groups SU and WI clustered individually

**Supplementary Material 6**: Gene function annotation results based on level 1 and 2 in KEGG database

**Supplementary Material 7**: Soil environmental factors detection and statistical test results. (* means significant difference(p < 0.05), ** means extremely significant difference (p < 0.01).)

**Supplementary Material 8**

## Data Availability

The raw data presented in this study can be found in National Center for Biotechnology Information (NCBI) Sequence Read Archive (SRA) data base through the accession number PRJNA1027241.
