# Supplementary figures and images for: Seasonal changes of soil microbiota and its association with environmental factors in coal mining subsidence area
Source: AMB Express. 2023 Dec 20;13:147. doi: 10.1186/s13568-023-01653-5 (PMC10733236; doi:10.1186/s13568-023-01653-5)

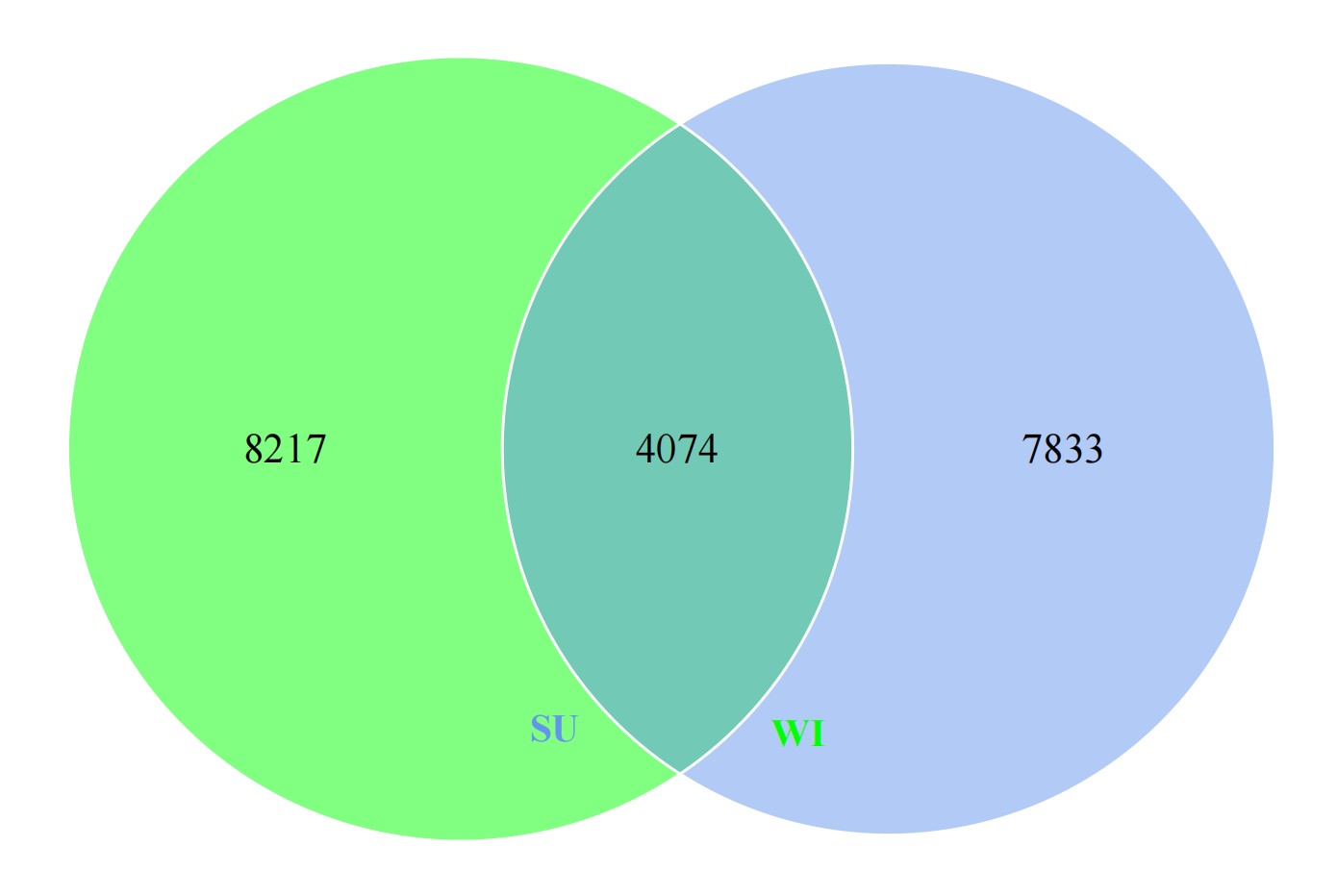

Supplement: Supplementary file 1 — Supplementary Material 1: Venn diagram shows number of genes common and unique to the groups SU and WI [file 13568_2023_1653_MOESM1_ESM.jpg]

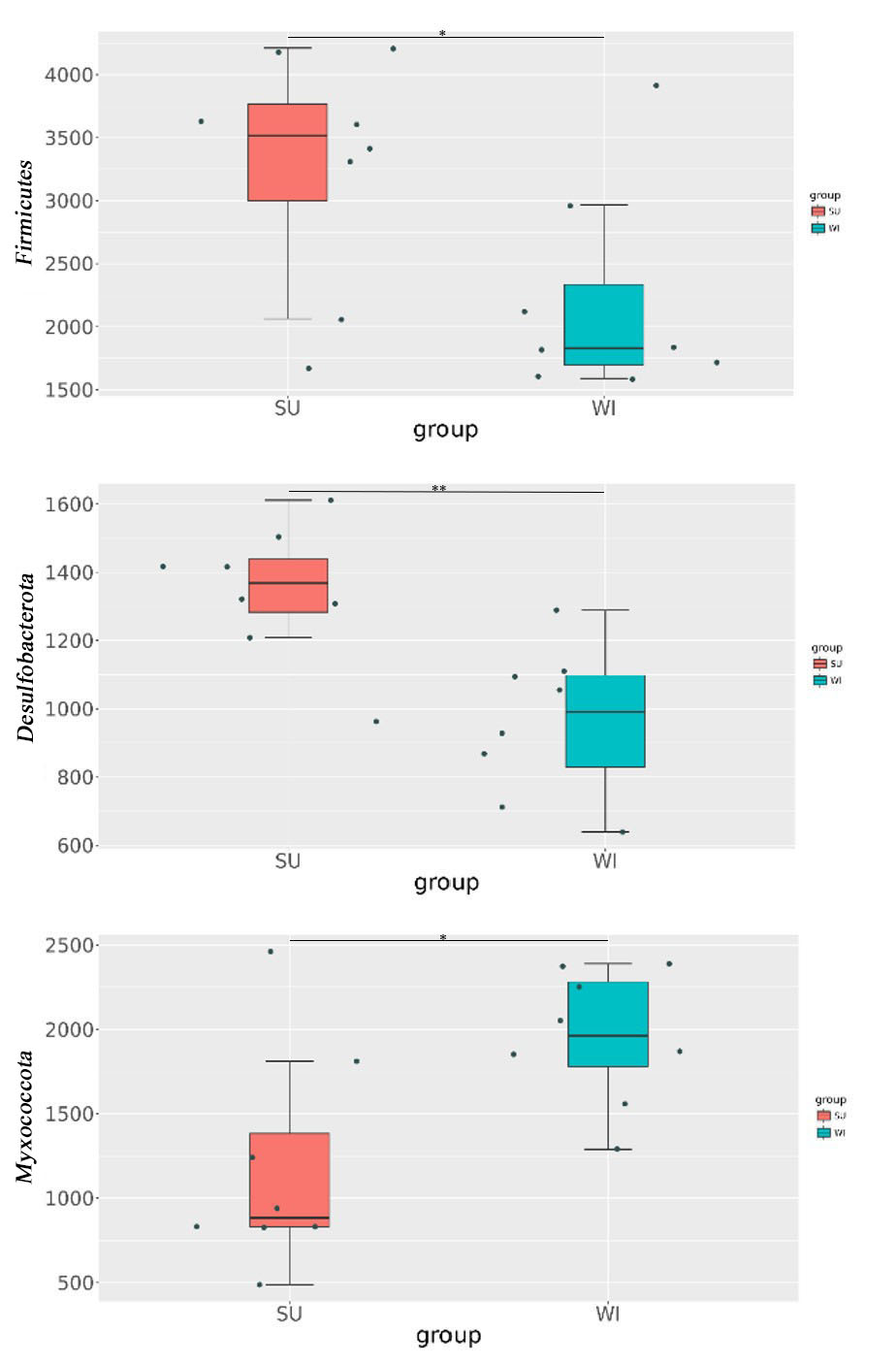

Supplement: Supplementary file 2 — Supplementary Material 2: Results of Metastat test at the phylum level. * means significant difference (p < 0.05), ** means extremely significant difference (p < 0.01) [file 13568_2023_1653_MOESM2_ESM.jpg]

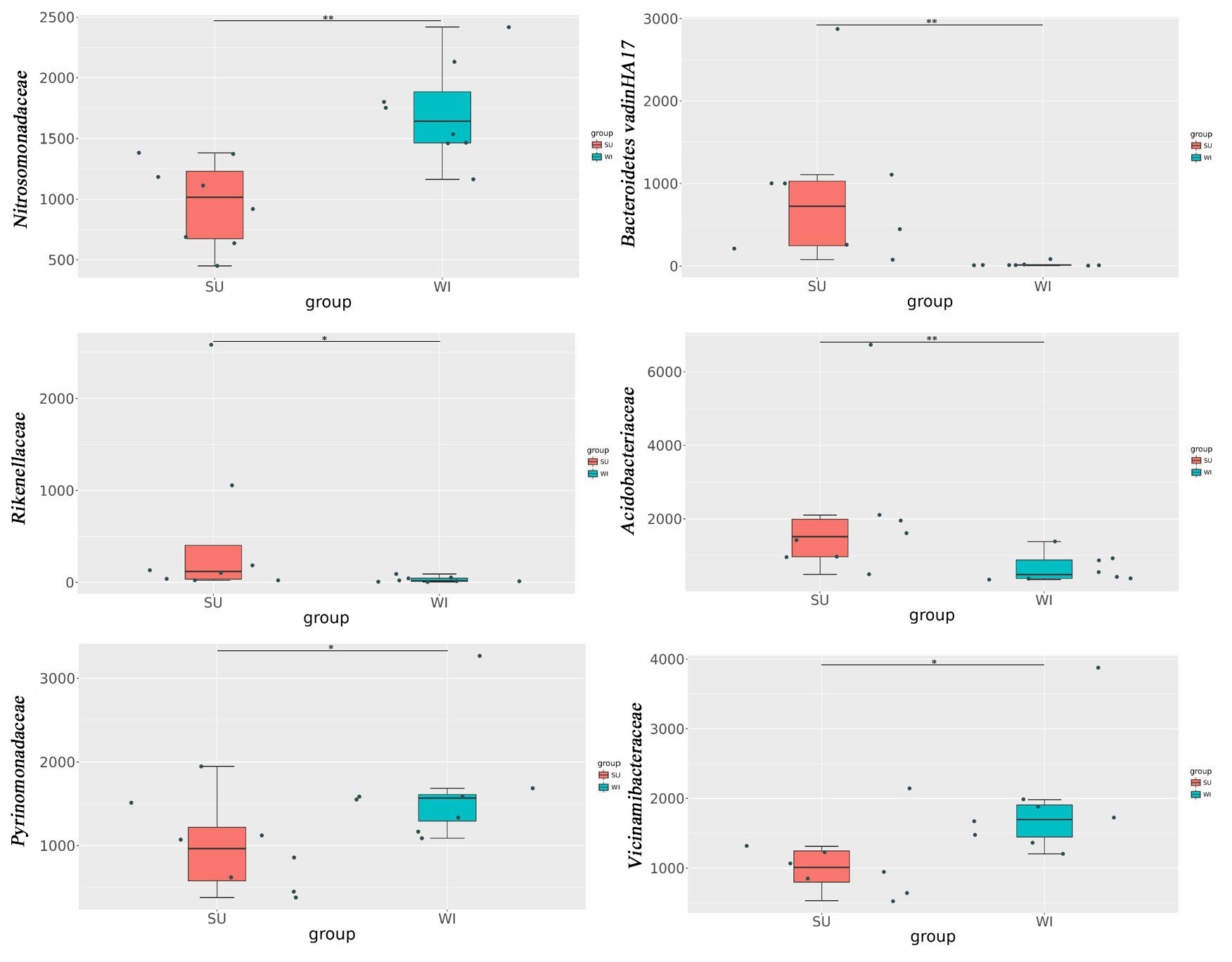

Supplement: Supplementary file 3 — Supplementary Material 3: Results of Metastat test at the family level. * means significant difference (p < 0.05), ** means extremely significant difference (p < 0.01) [file 13568_2023_1653_MOESM3_ESM.jpg]

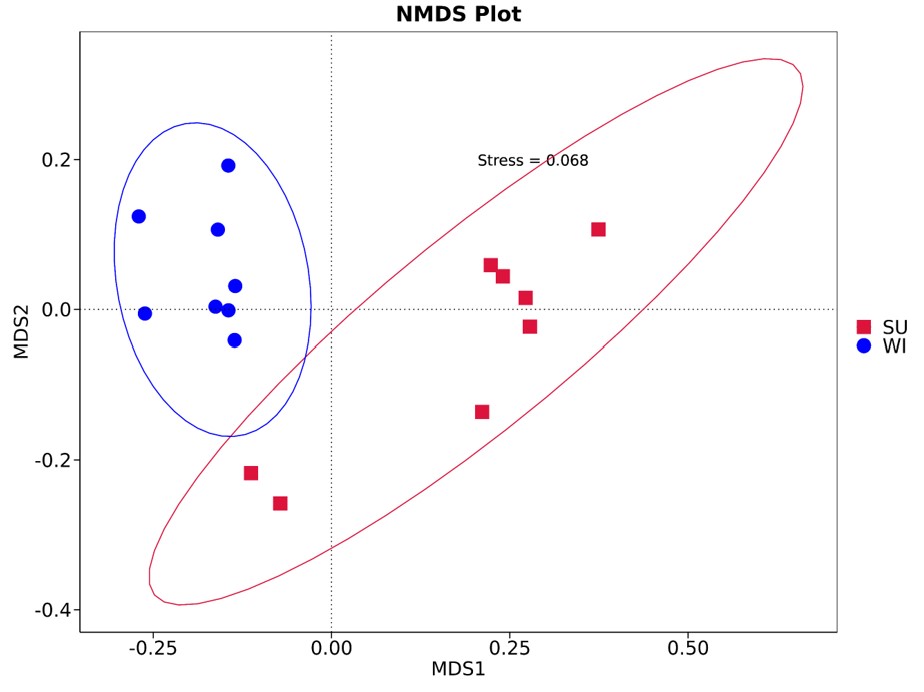

Supplement: Supplementary file 4 — Supplementary Material 4: PCOA analysis based on the abundance of annotated genes showed that samples from the groups SU and WI clustered individually [file 13568_2023_1653_MOESM4_ESM.jpg]

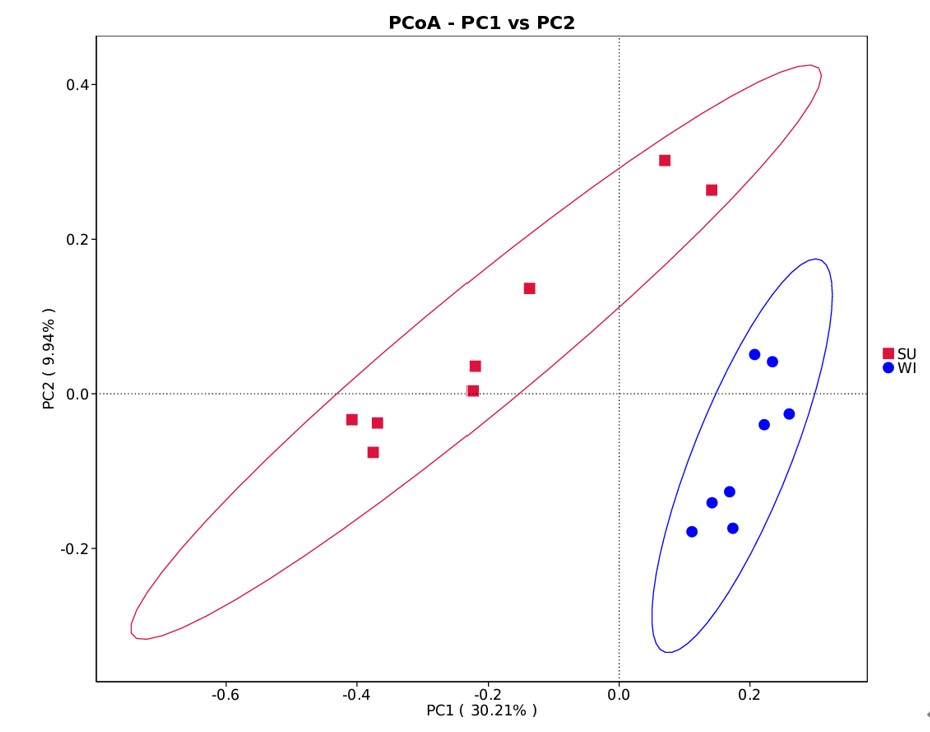

Supplement: Supplementary file 5 — Supplementary Material 5: NMDS analysis based on the abundance of annotated genes showed that samples from the groups SU and WI clustered individually [file 13568_2023_1653_MOESM5_ESM.jpg]

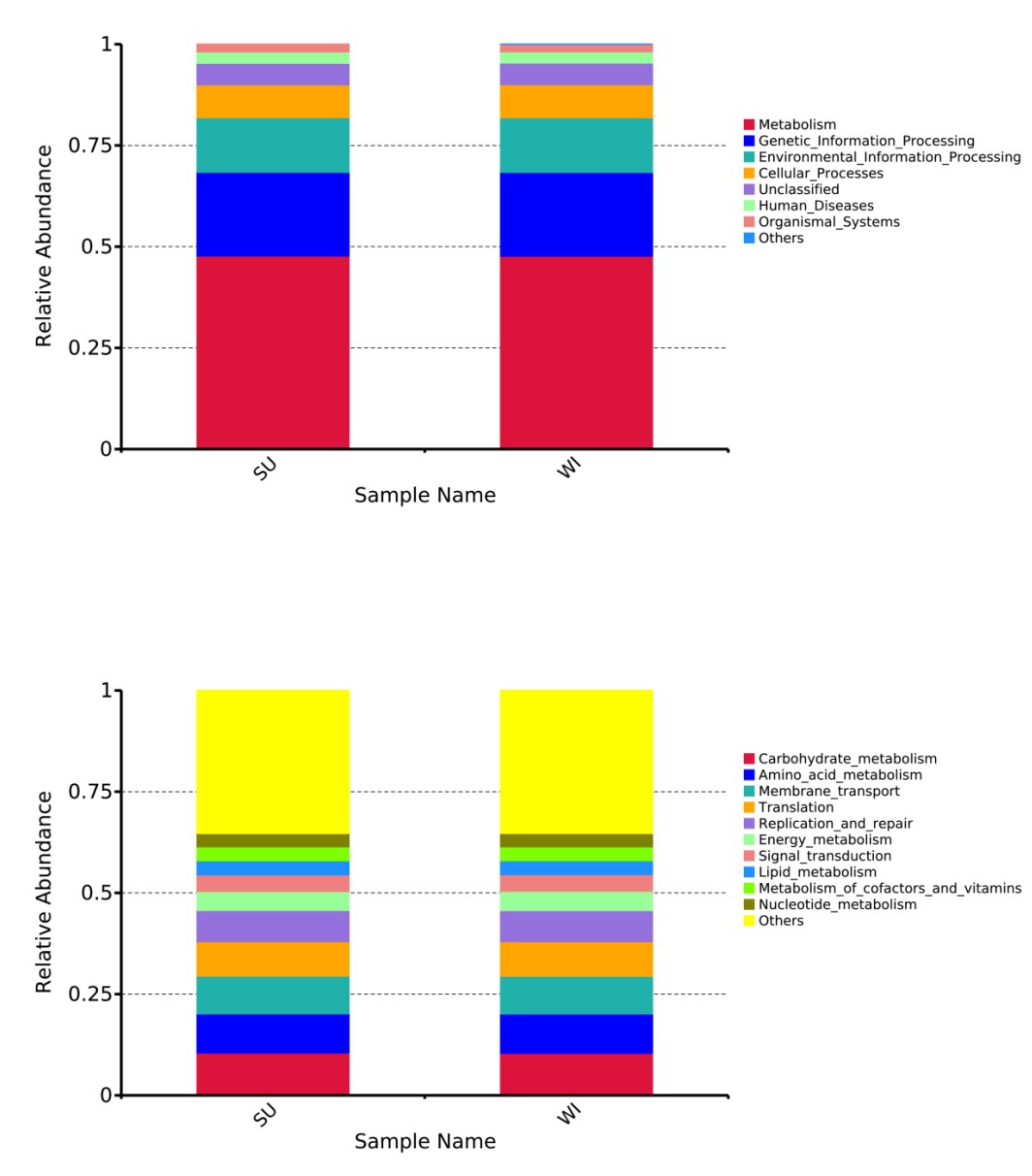

Supplement: Supplementary file 8 — Supplementary Material 8 [file 13568_2023_1653_MOESM8_ESM.jpg]
