# Supplementary material for: Seasonal changes of soil microbiota and its association with environmental factors in coal mining subsidence area
Source: AMB Express. 2023 Dec 20;13:147. doi: 10.1186/s13568-023-01653-5 (PMC10733236; doi:10.1186/s13568-023-01653-5)
Supplement: Supplementary file 6 — Supplementary Material 6: Gene function annotation results based on level 1 and 2 in KEGG database [file 13568_2023_1653_MOESM6_ESM.docx]

**Supplementary material**

**Supplementary Figure 1**

Venn diagram shows number of genes common and unique to the groups SU and WI.

**Supplementary Figure 2**

Results of Metastat test at the phylum level. “*” means significant difference(p<0.05), “**” means extremely significant difference (p<0.01).

**Supplementary Figure 3**

Results of Metastat test at the family level. “*” means significant difference(p<0.05), “**” means extremely significant difference (p<0.01).

**Supplementary Figure 4**

PCOA analysis based on the abundance of annotated genes showed that samples from the groups SU and WI clustered individually.

**Supplementary Figure 5**

NMDS analysis based on the abundance of annotated genes showed that samples from the groups SU and WI clustered individually.

**Supplementary Figure 6**

Gene function annotation results based on level 1 and 2 in KEGG database.
