# Supplementary material for: Seasonal changes of soil microbiota and its association with environmental factors in coal mining subsidence area
Source: AMB Express. 2023 Dec 20;13:147. doi: 10.1186/s13568-023-01653-5 (PMC10733236; doi:10.1186/s13568-023-01653-5)
Supplement: Supplementary file 7 — Supplementary Material 7: Soil environmental factors detection and statistical test results. (* means significant difference(p < 0.05), ** means extremely significant difference (p < 0.01).) [file 13568_2023_1653_MOESM7_ESM.docx]

Supplementary Table 1. Soil environmental factors detection and statistical test results

| Sample | Temp | PH | TP | TOC | AP | MC | DMC | TN |
| --- | --- | --- | --- | --- | --- | --- | --- | --- |
| SU1 | 24.80 | 7.23 | 69.86 | 7.58 | 4.59 | 0.36 | 0.97 | 1065.19 |
| SU2 | 26.60 | 7.34 | 65.86 | 7.83 | 3.02 | 0.34 | 0.97 | 1284.32 |
| SU3 | 26.50 | 6.74 | 80.48 | 8.24 | 6.75 | 0.36 | 0.98 | 693.90 |
| SU4 | 25.80 | 7.35 | 74.83 | 7.35 | 4.47 | 0.47 | 0.98 | 1539.96 |
| SU5 | 27.60 | 7.50 | 74.96 | 6.68 | 3.81 | 0.36 | 0.96 | 724.33 |
| SU6 | 26.20 | 6.60 | 69.84 | 5.17 | 7.54 | 0.35 | 0.95 | 1533.87 |
| SU7 | 26.40 | 6.96 | 58.62 | 6.80 | 2.30 | 0.30 | 0.96 | 535.64 |
| SU8 | 29.00 | 5.72 | 68.48 | 7.91 | 4.86 | 0.32 | 0.98 | 973.89 |
| WI1 | 8.30 | 6.71 | 0.14 | 13.59 | 0.23 | 0.37 | 0.98 | 1512.50 |
| WI2 | 8.30 | 6.15 | 0.15 | 13.66 | 0.33 | 0.27 | 0.99 | 1695.00 |
| WI3 | 7.50 | 6.80 | 0.15 | 12.53 | 0.37 | 0.25 | 0.99 | 970.00 |
| WI4 | 5.70 | 6.94 | 0.12 | 14.28 | 0.39 | 0.25 | 0.98 | 760.00 |
| WI5 | 7.10 | 7.23 | 0.15 | 22.27 | 0.26 | 0.29 | 0.98 | 367.50 |
| WI6 | 9.10 | 7.33 | 0.15 | 19.15 | 0.24 | 0.38 | 0.98 | 1995.00 |
| WI7 | 10.80 | 5.80 | 0.16 | 10.51 | 0.30 | 0.37 | 1.00 | 1895.00 |
| WI8 | 10.30 | 6.89 | 0.15 | 10.5 | 0.21 | 0.35 | 0.99 | 1590.00 |
| p-value | ** | 0.4828 | ** | ** | ** | 0.11 | ** | 0.2377 |

(“*” means significant difference(p<0.05), “**” means extremely significant difference (p<0.01).)
